# IL-10 and NOS2 Modulate Antigen-Specific Reactivity and Nerve Infiltration by T Cells in Experimental Leprosy

**DOI:** 10.1371/journal.pntd.0003149

**Published:** 2014-09-11

**Authors:** Deanna A. Hagge, David M. Scollard, Nashone A. Ray, Vilma T. Marks, Angelina T. Deming, John S. Spencer, Linda B. Adams

**Affiliations:** 1 Department of Health and Human Services, Health Resources and Services Administration, Healthcare Systems Bureau, National Hansen's Disease Programs, Baton Rouge, Louisiana, United States of America; 2 Mycobacterial Research Laboratories, Anandaban Hospital, Kathmandu, Nepal; 3 Colorado State University, Ft. Collins, Colorado, United States of America; University of Tennessee, United States of America

## Abstract

**Background:**

Although immunopathology dictates clinical outcome in leprosy, the dynamics of early and chronic infection are poorly defined. In the tuberculoid region of the spectrum, *Mycobacterium leprae* growth is restricted yet a severe granulomatous lesion can occur. The evolution and maintenance of chronic inflammatory processes like those observed in the leprosy granuloma involve an ongoing network of communications via cytokines. IL-10 has immunosuppressive properties and IL-10 genetic variants have been associated with leprosy development and reactions.

**Methodology/Principal Findings:**

The role of IL-10 in resistance and inflammation in leprosy was investigated using *Mycobacterium leprae* infection of mice deficient in IL-10 (IL-10−/−), as well as mice deficient in both inducible nitric oxide synthase (NOS2−/−) and IL-10 (10NOS2−/−). Although a lack of IL-10 did not affect *M. leprae* multiplication in the footpads (FP), inflammation increased from C57Bl/6 (B6)<IL-10−/−<NOS2−/−<10NOS2−/−. While IL-10−/− mice exhibited modest FP induration compared to B6, NOS2−/− and 10NOS2−/− mice developed markedly enlarged FP marking distinct phases: early (1 month), peak (3–4 months), and chronic (8 months). IFN-γ-producing CD4+CD44+ cells responding to *M. leprae* cell wall, membrane, and cytosol antigens and ML2028 (Ag85B) were significantly increased in the evolved granuloma in NOS2−/− FP compared to B6 and IL-10−/− during early and peak phases. In 10NOS2−/− FP, CD4+CD44+ and especially CD8+CD44+ responses were augmented even further to these antigens as well as to ML0380 (GroES), ML2038 (bacterioferritin), and ML1877 (EF-Tu). Moreover, fragmented nerves containing CD4+ cells were present in 10NOS2−/− FP.

**Conclusions/Significance:**

The 10NOS2−/− strain offers insight on the regulation of granuloma formation and maintenance by immune modulators in the resistant forms of leprosy and presents a new model for investigating the pathogenesis of neurological involvement.

## Author Summary

Despite effective antimicrobial therapy, 30–50% of leprosy patients develop immunological complications called leprosy reactions before, during or even years after being cured. Leprosy reactions are a major risk for neuritis that leads to peripheral nerve damage, disfigurement and disability. Unfortunately, why and how leprosy reactions occur is not well understood. Based on the latest human genetic leprosy susceptibility research and mouse infection models, we generated a double knockout mouse strain (10NOS2−/−) which has deficiencies in two key immune factors, interleukin-10 (IL-10) and inducible nitric oxide synthase (NOS2). We investigated the dynamics of the immune response to *Mycobacterium leprae* infection and chronicled the types of immune cells recruited to the site of infection. 10NOS2−/− mice developed a substantial induration in response to infection, as well as an increased interferon-gamma response to components of the leprosy bacillus. Interestingly, these animals also exhibited CD4+ T cell infiltration into the nerves, a phenomenon which has not been previously reported in leprosy mouse models. This new model provides insight into potential mechanisms whereby immune modulators may regulate leprosy reactions and neuritis and could aid the development of tests for monitoring and treatment of leprosy patients.

## Introduction

Leprosy is a neglected tropical disease that is still diagnosed in >200,000 new patients every year [1]. Its clinical spectrum is associated with a diverse and often dynamic immune response ranging from strong cell mediated immunity (CMI) at one end to complete anergy toward *Mycobacterium leprae* antigens at the other. As patients are often not diagnosed until years post-infection, the early stage determinants of disease resolution or progression are not yet understood. Likewise, much remains unknown regarding the immunopathogenesis of leprosy neuropathy which can occur even after successful antimicrobial therapy. Several global research collaborations are actively endeavoring to develop effective vaccines and new diagnostic methods [2]–[10], but considerable additional effort is needed to ultimately eliminate leprosy.

The majority of leprosy patients are classified into the borderline area of the spectrum [11] where there appears to be a partial immunity of an undefined nature which allows neither complete anergy nor resolution of disease. Borderline leprosy can be immunologically unstable, permitting upgrading and downgrading responses due to immunological fluctuations or acute reactional episodes that may cause significant tissue destruction. In an effort to investigate this broad range of responses within the lesion, we have evaluated the *M. leprae-*induced footpad (FP) granuloma in a number of mouse strains with immune defects [12]–[16], including inducible nitric oxide synthase knockout mice (NOS2−/−) [17], [18]. NOS2−/− mice respond to *M. leprae* infection in a manner that resembles borderline tuberculoid disease in that bacterial growth is restricted and they develop a large granulomatous response, composed of epithelioid macrophages and numerous lymphocytes, which infiltrates surrounding tissue.

IL-10 is an anti-inflammatory and immunosuppressive cytokine produced primarily by macrophages and T cells. IL-10 polymorphisms have been associated with leprosy resistance or susceptibility in several endemic populations [19]–[25], and variations in IL-10 expression have been noted in relation to the occurrence and treatment of reactions [26]–[28]. In addition, Toll-like receptor 2 polymorphisms have been linked to susceptibility and increased production of IL-10 [29]–[31]. Therefore, IL-10 appears to play a major role in the course of this chronic infectious disease although the precise mechanisms of action within the site of infection are unknown. A better understanding of its involvement could benefit our ability to control the pathological consequences of leprosy.

Based upon these collective observations, we hypothesized that mice having an IL-10 deficiency would exhibit a more robust immune response toward *M. leprae*. Furthermore, a lack of IL-10 in the absence of NOS2 would further intensify the NOS2 immunopathological response, conceivably driving that model toward a more inflammatory or “reactional” state. Therefore, we examined *M. leprae* infection in IL-10−/− and NOS2−/− mice, as well as double knockout mice (10NOS2−/−), and evaluated three issues directly in the FP granulomas over the course of long term infection: 1) growth of the bacilli and histopathology, 2) host cellular dynamics evoked by *M. leprae* infection in the FP lesion, and 3) characterization of *M. leprae* antigen-specific T cells. Results show that while NOS2 is largely responsible for regulating the magnitude of T cell infiltration into the granuloma, a concomitant lack of IL-10 results in intensified *M. leprae* antigen responsiveness and T cell invasion of nerves.

## Methods

### Ethics statement

These studies were performed under a scientific protocol reviewed and approved by the National Hansen's Disease Programs Institutional Animal Care and Use Committee (Assurance #A3032-01), and were conducted in accordance with all state and federal laws in adherence with PHS policy and as outlined in *The Guide for the Care and Use of Laboratory Animals, Eighth Edition*.

### Maintenance of viable *M. leprae* inoculum

*M. leprae* strain Thai-53 was propagated in athymic *nu/nu* mice (Harlan Sprague-Dawley, Inc., Indianapolis, IN). *M. leprae* were harvested from the FP and viability was assessed by radiorespirometry, which measures the oxidation of ^14^C-palmitic acid to ^14^CO_2_ [32] and vital staining, which utilizes Syto9 and propidium iodide to determine cell wall integrity (Bacterial Viability Staining Kit, Life Technologies, Grand Island, NY) [33]. *M. leprae* bacilli were stored at 4°C and used within 24 hours of harvest.

### Mice

Female mice 5–7 weeks old were obtained from Jackson Laboratories (Bar Harbor, ME) from the following strains: IL-10−/− (B6.129P2-*IL-10* ^tm1Cgn/J^), NOS2−/− (B6.129P2-*NOS2* ^tm1Lau/J^) and C57BL/6J mice (B6). IL-10−/−/NOS2−/− double knockout mice (10NOS2−/−) were generated by crossing IL-10−/− and NOS2−/− strains to produce offspring bearing both knockout mutations. Mice were housed under pathogen-free conditions in laminar flow animal isolators, in sterile cages, and maintained on sterile food and water. Like the parental strains, the 10NOS2−/− mice thrived in these conditions and were good breeders. They exhibited no overt differences in phenotypic or clinical features.

### *M. leprae* growth assay

Mice were infected with freshly harvested, viable *M. leprae* via inoculation of a low dose of 6×10^3^ bacilli in 0.03 ml phosphate buffered saline (PBS) into each hind FP. To assess bacterial multiplication, *M. leprae* were harvested from the FP and acid fast bacilli (AFB) were counted as previously described [32]. *M. leprae* were enumerated at 3 months to verify early bacterial growth, at 6 months because maximum growth is reached in the control strain at this time, and at 12 months to check for clearance or continued growth in the knockout strains. FP tissue was examined by histology.

### Histopathology

Feet were fixed in 10% buffered formalin, decalcified in 10% (w/v) sodium citrate and 22.5% (v/v) formic acid, and embedded in paraffin [15]. Cross-sections (4 µm) from the distal, mid and proximal areas of the metatarsus were stained with hematoxylin and eosin. An Olympus BX53 microscope (Center Valley, PA) equipped with a Retiga 2000R Firewire Digital camera and cellSens Imaging Software was used to assess and capture images from the stained tissues.

### FP granuloma and induration assay

Mice were infected with *M. leprae* via inoculation of a high dose of 3×10^7^ bacilli in 0.03 ml PBS into each hind FP [14], [15]. Induration was measured weekly using a Vernier caliper. To characterize the granulomatous response, FP were harvested at 1 month (early), 4 months (peak induration), and 8 months (chronic) post-infection and evaluated for cell phenotypes, cytokine expression, and *M. leprae* antigen responsiveness.

### Immunohistochemistry

O.C.T. medium (Tissue Tek, Inc., Torrence, CA) embedded FP tissue was snap frozen in liquid nitrogen and stored at −70°C. Serial 4 µm sections (Frigocut 2800E, Leica Microsystem, Inc., Bannockburn, IL) were fixed in cold acetone and, after blocking with avidin and biotin (Vector Laboratories, Burlingame, CA), stained with rat anti-mouse CD4 (BD Biosciences, San Jose, CA). Biotin-labeled mouse anti-rat F(ab)_2_ fragments (Jackson ImmunoResearch Laboratories, Inc., West Grove, PA) was applied, and immunohistochemical visualization was achieved with Vectastain *Elite* ABC kit (Vector), the AEC Substrate kit (Vector), and hematoxylin counterstain.

### Isolation of total RNA and preparation of cDNA

RNA was prepared from FP tissue of individual mice as described previously [15]. cDNA was generated from 0.5 µg RNA using random hexamers and an RT-for-PCR kit (Clontech, Palo Alto, CA) at 42°C for 1 hr in a thermocycler (9600, Perkin-Elmer Corp., Norwalk, CT). Controls for DNA contamination were prepared from RNA samples using the reverse transcription reagents minus the reverse transcriptase. All sample and control preparations were aliquoted and stored at −70°C.

### Real-time reverse transcription-polymerase chain reaction (RT-PCR)

Real-time RT-PCR for IFN-γ transcripts were carried out via Taqman technology using specific primer sets and probes and Universal Master Mix (Applied BioSystems) in an ABI PRISM 7300 Sequence Detection System (Applied BioSystems). Semiquantitative analysis of the data was performed using the ΔΔC_T_ method and expressed as a fold increase in cytokine expression over uninfected FP. Data were normalized for template variation using the GAPDH RT-PCR value for the same template.

### *M. leprae* viability assay

FP tissue, which was fixed in 70% ethanol and stored at −20°C until processing, was rehydrated in water and suspended in TRIzol reagent. RNA and DNA were extracted using the FastPrep FP 24 instrument (MP Biomedicals, Solon, OH) as described previously [34], [35]. The number of *M. leprae* was determined on the DNA fraction of each specimen via Taqman methodology using the standard curve method, primers, and a probe specific for a common region of the repetitive element, RLEP [36]. Based on this RLEP count, the RNA equivalent of 3×10^3^ *M. leprae* from the purified RNA fraction was converted to cDNA using an Advantage RT-for-PCR kit (Clontech, Mountain View, CA). As a control for possible DNA contamination, “mock” cDNA was prepared using an equivalent amount of RNA, polymerase mix, and primers without the reverse transcriptase. The viability of the *M. leprae* in each sample was determined on cDNA, using Taqman technology, the standard curve method, and primers and probes specific for *esxA* (encodes the ESAT-6 protein) [35].

### Flow cytometry

FP tissues were aseptically minced and digested using collagenase and DNase to generate single cell suspensions as previously described [37]. Cells were counted, treated with Fc Block (CD16/CD32[Fcγ III/II receptor]; BD Biosciences) and stained with the appropriate isotype control antibodies or one or more of the following: anti-CD3 (clone 17A2), anti-CD4 (clone RM4-5), anti-CD8a (clone 53-6.7), anti-CD44 (clone IM7), anti-CD244.2 (clone 2B4), anti-CD11b (clone M1/70), anti-I-A^b^ (clone 25-9-17), and anti-Ly-6G (clone 1A8) (BD Biosciences). Data was collected using a FACS Aria interfaced with Hewlett Packard 4100 running FACS Diva software (BD Biosciences).

### Intracellular flow cytometry

FP cells were enriched for lymphocytes by adherence and non-adherent cells were plated in 96 well plates at 2×10^5^ cells in 200 µl medium (RPMI 1640 [Life Technologies], 10% fetal bovine serum [HyClone Laboratories, Logden, UT], 25 mM HEPES buffer [Life Technologies], 0.2% NaHCO_3_ [Life Technologies], 2 mM glutamine [Irvine Scientific, Santa Ana, CA], 100 µg/ml ampicillin [Sigma-Aldrich, St. Louis, MO]) containing 1×10^−5^ M 2-mercaptoethanol (Sigma-Aldrich). Cells were incubated overnight at 33°C with PBS (Irvine Scientific), anti-CD3 (BD Biosciences) or 10 µg/ml of the following *M. leprae* antigens: Membrane, Cell wall antigen (CWA), Cytosol, and five recombinant proteins which previously had been shown to be immunologically important and/or recognized by either cell mediated or serological responses in leprosy patients [9], [10]: ML2028 (Ag85B, belongs to the mycolyltransferase Ag85 family and is an essential enzyme involved in cell wall biogenesis [38]), ML0050 (CFP-10, culture filtrate protein which can elicit potent early cell mediated immune responses), ML0380 (GroES, a chaperonin protein involved in protein folding, it is one of the most highly expressed native proteins of the leprosy bacillus [39] and is recognized by one third of all *M. leprae* reactive T cells in tuberculoid leprosy patients and healthy household contacts [40]), ML2038 (BfrA, bacterioferritin is a membrane protein involved in iron uptake [41]), and ML1877 (EF-Tu, elongation factor involved in protein synthesis). These antigens were provided through the NIH/NIAID Leprosy Research Contract N01 AI-25469 from Colorado State University and are currently supplied through BEI Resources (Manassas, VA). Cells were collected and stained for CD4, CD8, CD44 and intracellular IFN-γ (clone XMG1.2) using BD Cytofix/Cytoperm Plus Kit with BD GolgiPlug according to kit instructions.

### Bioplex assay for cytokine and chemokine determination

Cytokine and chemokine concentrations in the supernatants were determined using a Bioplex Mouse Cyto 23Plex Kit and analyzed on the Bio-Plex System with Luminex xMap Technology (Life Science Research, Hercules, CA). Analytes targeted were: IFN-γ, IL-1α, IL-1β, IL-2, IL-3, IL-4, IL-5, IL-6, IL-9, IL-10, IL-12p40, IL-12p70, IL-13, IL-17, Eotaxin, KC, G-CSF, GM-CSF, TNF, CCL-2, CCL-3, CCL-4, and CCL-5.

### Statistical analyses

Growth of *M. leprae* was analyzed using the GLM procedure (SAS 9.3) with an analysis of variance in a factorial arrangement of group vs. time, followed by post hoc comparisons with pairwise *t*-tests of least square means and non-parametric Mann-Whitney tests. All other data were analyzed using unpaired *t*-tests with or without Welch's correction (SigmaPlot 12.0, SyStat Software). Data were considered significant at *p*≤0.05 [14], [15].

## Results

### Effects of NOS2 deficiency on *M. leprae* growth and induration

**Figure 1:**
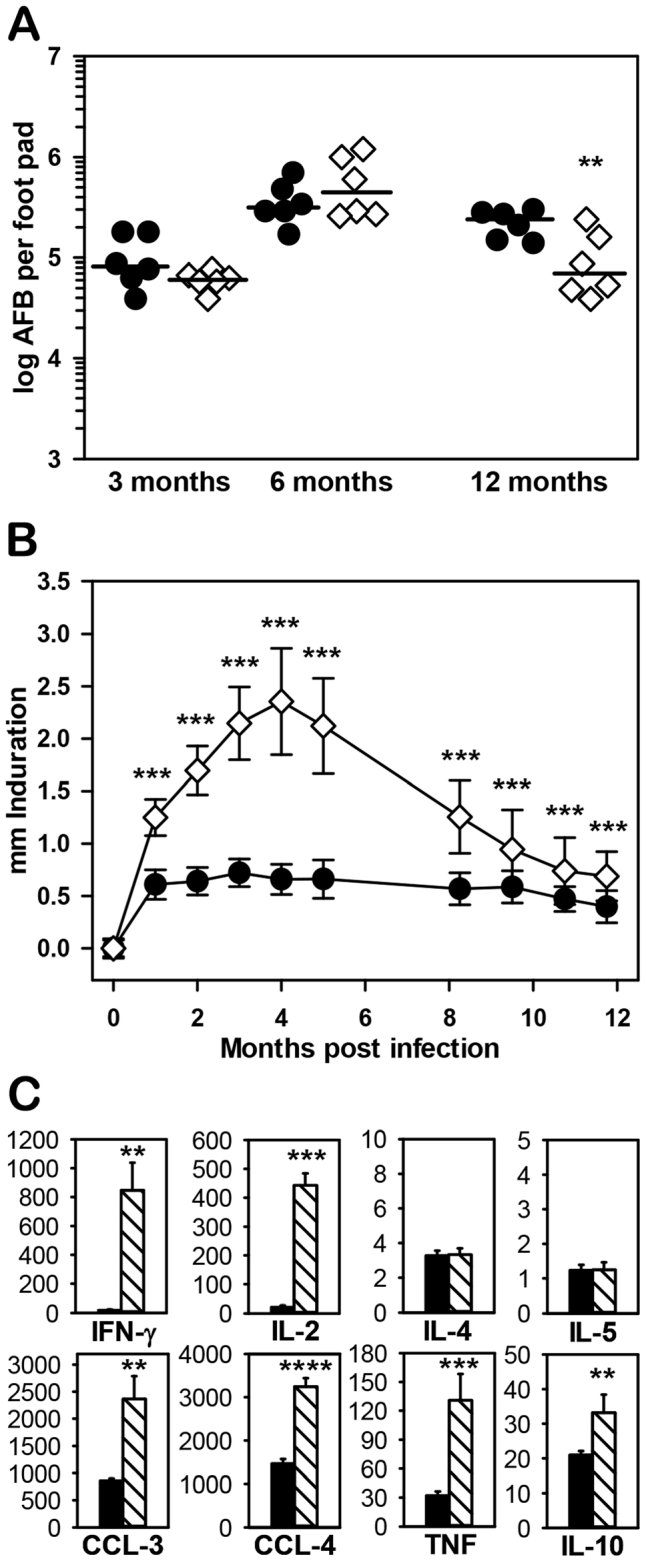
Response of NOS2−/− mice to infection with *M. leprae*. (A) Growth of *M. leprae* was determined in B6 (black circles) and NOS2−/− (diamonds) mice infected in each hind FP with 6×10^3^ *M. leprae*. At 3, 6, and 12 months, the number of AFB per FP was determined. Each symbol represents one mouse. The threshold of counting ability is 4.8×10^3^ AFB per FP. B6 vs. NOS2−/− at 3 months (*p* = 0.1885), 6 months (*p* = 0.2516), and 12 months (*p* = 0.0049). (B) B6 (black circles) and NOS2−/− (diamonds) mice were inoculated with 3×10^7^ *M. leprae* in each hind FP and FP induration was measured monthly using a Vernier caliper. (C) FP granuloma cells were harvested from B6 (black bars) and NOS2−/− (striped bars) mice 4 months post inoculation with 3×10^7^ *M. leprae*. Non-adherent cells were plated at 2×10^5^ cells per well and stimulated with *M. leprae* membrane antigen for 48 hr. Supernatants were collected and tested for cytokine and chemokine production by Bioplex assay. Experiments shown are representative of at least two independent experiments. *p≤0.05; **p≤0.01; ***p≤0.001.

We first assessed growth of *M. leprae* in NOS2−/− mice using the standard mouse FP growth assay, which exploits the fact that a minor inoculum of *M. leprae* (≤10^4^) into the FP of immunocompetent mice can initially evade immune-mediated killing, grow locally for approximately 6 months, and peak at 10^5^–10^6^ bacilli when growth is halted by the immune response [42]. As shown in Fig. 1A, multiplication of the bacilli was similar in the B6 and NOS2−/− strains and peaked on the order of 10^5^ AFB per FP by 6 months post infection. Extended observation demonstrated that *M. leprae* growth did not continue past the 6 months peak in the NOS2−/− but showed a trend toward improved clearance at 12 months (*p* = 0.0049).

**Figure 2:**
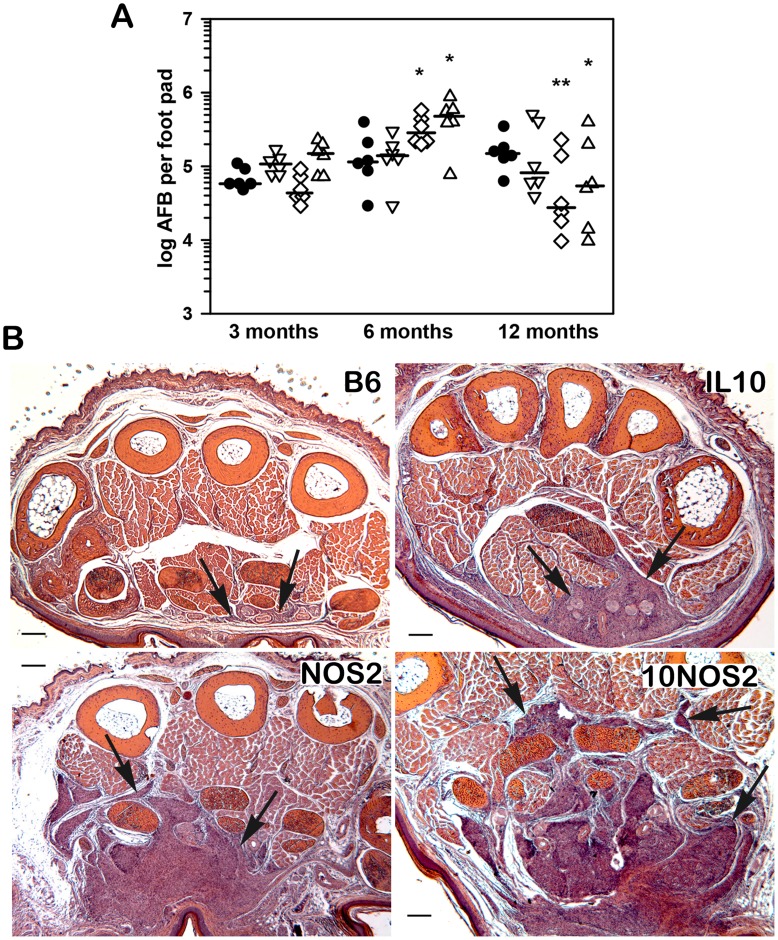
Growth of *M. leprae* and granulomatous response in infected FP. (A) *M. leprae* shows a similar growth profile in control mice and mice with single or combined deficiencies in IL-10 or NOS2. B6 (black circles), IL-10−/− (inverted triangles), NOS2−/− (diamonds), and 10NOS2−/− (triangles) mice were infected in each hind FP with 6×10^3^ viable *M. leprae*. At 3, 6, and 12 months, the number of AFB per FP was determined. Each symbol represents one mouse. The threshold of counting ability is 4.8×10^3^ AFB per FP. (B) Histological examination (hematoxylin and eosin stains) of *M. leprae*-infected FP at 6 months post infection. In B6 mice, minimal interstitial infiltrates of mononuclear cells can be seen between muscles in the ventral portion of the FP (bottom of figure). In IL-10−/− FP, more extensive mononuclear cell infiltrates are seen in the subcutis as well as greatly expanded granulomatous inflammation that has replaced approximately 25% of the total muscle mass. In NOS2−/− FP, granulomatous inflammation has replaced nearly 50% of the muscle mass, as is also seen in 10NOS2−/−. Experiment shown is representative of two independent experiments. *p≤0.05; **p≤0.01.

The *M. leprae* growth assay is limited in that the minimal infection site that develops in immunocompetent mice (see Fig. 2B, [B6]) is insufficient for in depth investigation of the granuloma. Therefore, we utilize the FP induration assay, where adequate numbers of *M. leprae* are administered in the initial inoculum to induce a granuloma which can then be monitored via induration and at the cellular level throughout chronic infection. As shown in Fig. 1B, B6 FP reached peak induration of 0.72±0.13 mm at 3 months post infection and maintained this level for several months. In contrast, induration in NOS2−/− FP was significantly augmented by 1 month (*p*<0.001), continued to increase until it peaked at 2.36±0.51 mm at 4 months, then declined over the next 8 months.

In order to investigate the immune modulators within the site of infection, FP lymphocytes were harvested at 4 months and stimulated with *M. leprae* membrane antigen in vitro. Striking differences between the B6 and NOS2−/− strains were seen in cytokine and chemokine production. Large amounts of Th1 cytokines (IFN-γ and IL-2), chemokines (CCL-3 and CCL-4), and TNF (Fig. 1C), as well as other analytes associated with inflammation (IL-1α, IL-1β, IL-3, IL-9, IL-13, IL-17, CCL-2 CCL-5, G-CSF and GM-CSF [data not shown]) were generated by the NOS2−/− cells compared to B6 FP cells. In contrast, similar levels of IL-6, IL-12p40, IL-12p70, KC, and eotaxin (data not shown) and very low levels of the Th2 cytokines, IL-4 and IL-5 (Fig. 1C), were produced by both strains. Interestingly, significantly elevated levels of IL-10 (Fig. 1C) were generated by the NOS2−/− FP cells compared to B6 (*p* = 0.004).

Because IL-10 has immunosuppressive properties, we questioned whether its generation in the NOS2−/− mice may be an attempt to temper the inflammatory response generated at the site of infection. If so, we postulated that inhibiting both IL-10 and NOS2 could push the model to a more inflammatory state. Our initial attempts to inhibit both IL-10 and NOS2 involved supplementing the drinking water of *M. leprae*-infected IL-10−/− mice with L-NIL, a selective inhibitor of NOS2. These mice developed an enlarged FP induration over an 8 week period (data not shown). In order to perform longer term studies, however, we generated a NOS2 and IL-10 double knockout strain (10NOS2−/−) by cross-breeding mice with the individual knockouts.

### 10NOS2−/− limit growth of *M. leprae* in the FP but exhibit an enhanced magnitude in the granulomatous response

10NOS2−/−, alongside IL-10−/−, NOS2−/− and B6 mice, were inoculated with 6×10^3^ viable *M. leprae* in both hind FP and tissues were harvested at 3, 6 and 12 months post inoculation to assess growth of the bacilli. As shown in Fig. 2A, AFB counts from all knockout groups demonstrated a pattern of growth that was similar to the B6 strain in that they controlled infection and peaked at 6 months on the order of 10^5^ AFB per FP, albeit counts were somewhat higher in NOS2−/− (*p* = 0.0477) and 10NOS2−/− (*p* = 0.0146). Again, there was improved clearance in the NOS2−/− (*p* = 0.0069) at 12 months, as well as in the 10NOS2−/− (*p* = 0.0413). Although a restriction of growth of *M. leprae* was achieved by all groups, histological assessment indicated significant variations in the granulomatous responses (Fig. 2B). Mild interstitial mononuclear cell infiltrates were observed in B6 FP. More extensive cellular infiltration was observed in IL-10−/−, and the most extensive infiltrates were seen in NOS2−/− and 10NOS2−/−. In all strains, the infiltrates were composed of mononuclear cells, with few granulocytes. In both NOS2−/− and 10NOS2−/−, the inflammatory infiltrates replaced muscle bundles in the FP, but no active necrosis of muscle was seen and no epidermal changes were observed.

### Induration and IFN-γ expression in *M. leprae*-infected 10NOS2−/− FP over long term infection

**Figure 3:**
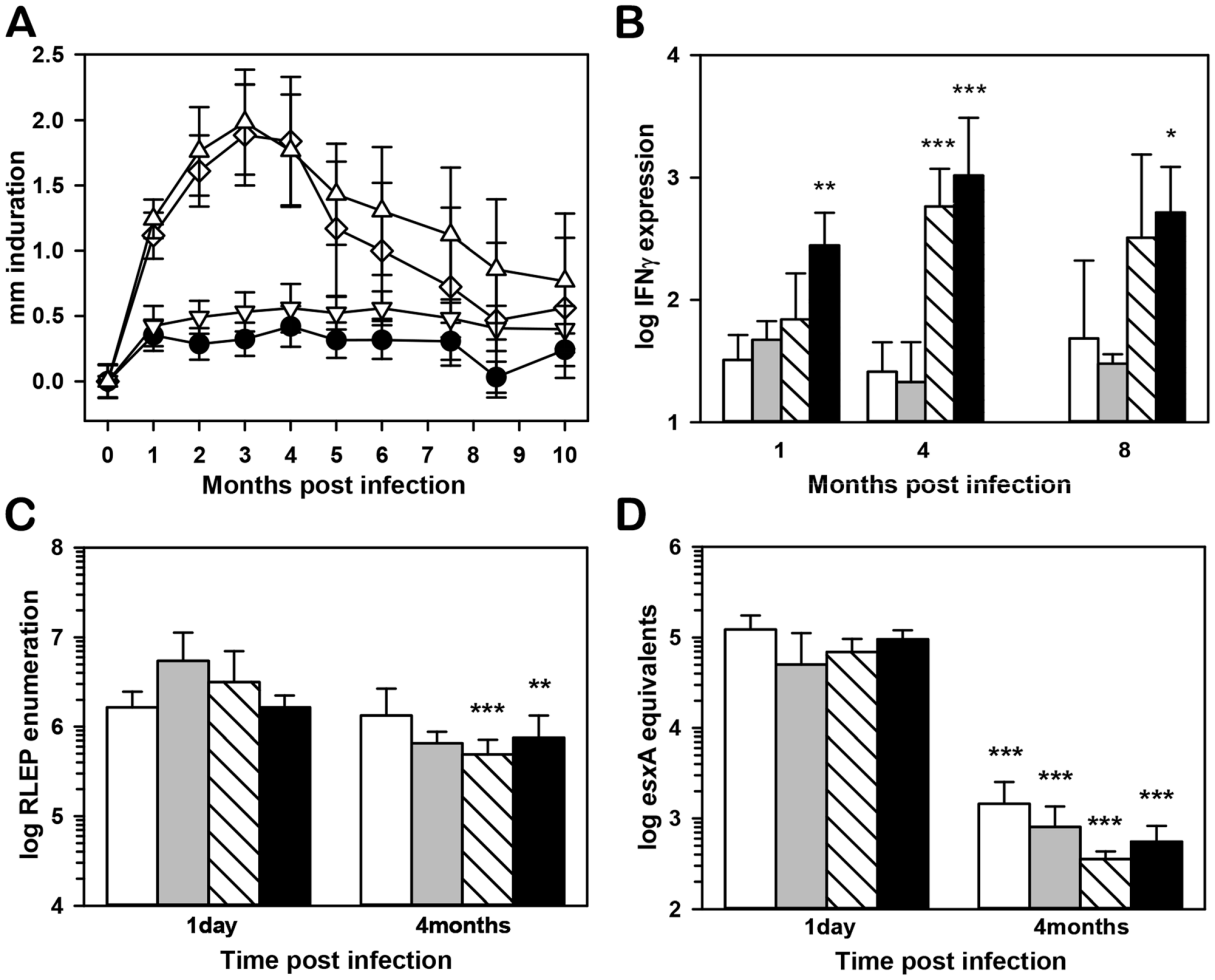
Induration, *M. leprae* viability, and IFN-γ expression in infected FP. (A) B6 (black circles), IL-10−/− (inverted triangles), NOS2−/− (diamonds), and 10NOS2−/− (triangles) FP were inoculated with 3×10^7^ *M. leprae* and FP induration was measured using a Vernier caliper. (B) IFN-γ expression in FP at 1, 4 and 8 months post-infection in B6 (white bars), IL-10−/− (gray bars), NOS2−/− (striped bars) and 10NOS2−/− (black bars). RNA was purified and subjected to real-time RT-PCR for IFN-γ. Gene expression was normalized to glyceraldehyde-3-phosphate dehydrogenase mRNA expression and reported as fold increase over uninfected FP. At 1 day and four months post infection, *M. leprae* per FP were (C) enumerated via RLEP Taqman and (D) bacterial viability was assessed by *esx*A RT-PCR in B6 (white bars), IL-10−/− (gray bars), NOS2−/− (striped bars) and 10NOS2−/− (black bars). Data shown are means +/− SD and are representative of two independent experiments. *p≤0.05; **p≤0.01; ***p≤0.001.

In order to further investigate granuloma dynamics, we assessed induration, cellular recruitment, IFN-γ expression, and bacterial viability over the course of long-term infection of the FP. As shown in Fig. 3A, induration in B6 FP reached a peak of approximately 0.5 mm at 1 month post infection and was maintained at this level for several months. FP thickness in IL-10−/− was greater than in B6 mice throughout infection (*p*<0.05). A strongly amplified pattern of induration was observed in NOS2−/− and 10NOS2−/− permitting division into three phases for investigation: early (1 month), peak (3–4 months) and chronic (>8 months). By 1 month, both NOS2−/− and 10NOS2−/− FP were significantly more indurated than either IL-10−/− or B6 FP (*p*<0.0001), and by peak phase both of these strains similarly exhibited >4 times the induration of IL-10−/− or B6 FP (*p*<0.0001). During the later phase of chronic infection, 10NOS2−/− showed a slower decline than NOS2−/− in FP induration and eventually diminished into a final plateau of approximately twice that of IL-10−/− and B6 FP (*p*<0.0001).

As shown in Fig. 3B, all strains demonstrated robust IFN-γ expression in response to *M. leprae* infection. B6 and IL-10−/− mice exhibited a >1 log increase in IFN-γ expression over uninfected FP throughout infection. In NOS2−/− FP, IFN-γ expression was increased more than 2 log over that in B6 FP at 4 months (*p*<0.001). Expression of IFN-γ was significantly greater in 10NOS2−/− FP compared to all other strains at 1 month, and this high level of expression was maintained throughout infection.

To verify that the augmented induration and IFN-γ expression seen in NOS2−/− and 10NOS2−/− FP was not due to enhanced bacterial growth in these strains, we determined *M. leprae* counts and viability in this high dose model. Compared to initial counts at 1 day, the number of *M. leprae* remained relatively constant in B6 and IL-10−/− FP or decreased slightly in NOS2−/− and 10NOS2−/− FP at 4 months post infection (Fig. 3C). Moreover, *M. leprae* exhibited high viability in all strains at 1 day (Fig. 3D) but bacterial viability declined significantly in all strains by 4 months, again indicating that the lack of NOS2 and/or IL-10 did not compromise immune-mediated killing of the bacilli.

### CD4+ T cell distribution in *M. leprae*-infected FP

**Figure 4:**
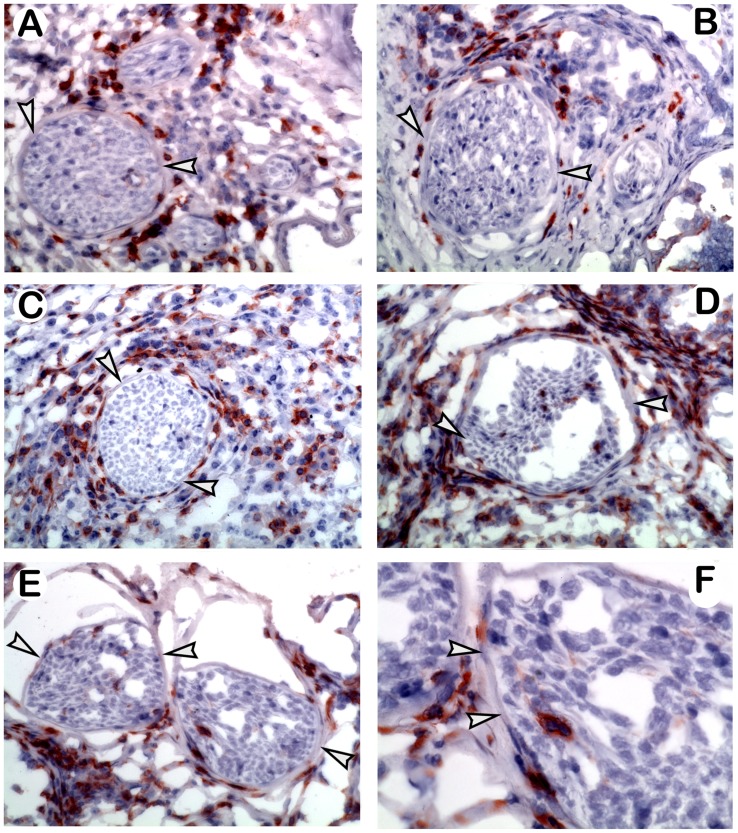
Endoneural CD4+ T cells in the *M. leprae*-infected 10NOS2−/− FP. Immunohistochemical staining of CD4+ T cells in FP at 4 months post *M. leprae* infection. Arrowheads indicate the perineural membrane. In (A) B6, (B) IL-10−/−, and (C) NOS2−/− mice, CD4+ T cells infiltrated the FP and surrounded but did not enter the nerves. In contrast, nerves in 10NOS2−/− FP (D–F) exhibited CD4+ T cells in the endoneurium. Experiment shown is representative of two independent experiments. A–E: 40× magnification; F: 60× magnification of nerve in Fig. E.

*M. leprae*-infected FP were evaluated at 4 months by immunohistochemistry for the presence and distribution of CD4+ T cells. As shown in Fig. 4A, CD4+ cells in B6 FP were distributed throughout the lesion and characteristically surrounded intact nerves. A similar pattern was seen in the IL-10−/− FP (Fig. 4B). NOS2−/− and 10NOS2−/− had substantially more CD4+ cells in the FP. In NOS2−/− FP (Fig. 4C), CD4+ cells surrounded the nerves in a pattern similar to B6 and IL-10−/−. In contrast, in 10NOS2−/− FP (Fig. 4D–F), CD4+ cells were observed inside fragmented nerves.

### NOS2−/− and 10NOS2−/− exhibit augmented T cell infiltration into the *M. leprae*-infected FP

**Figure 5:**
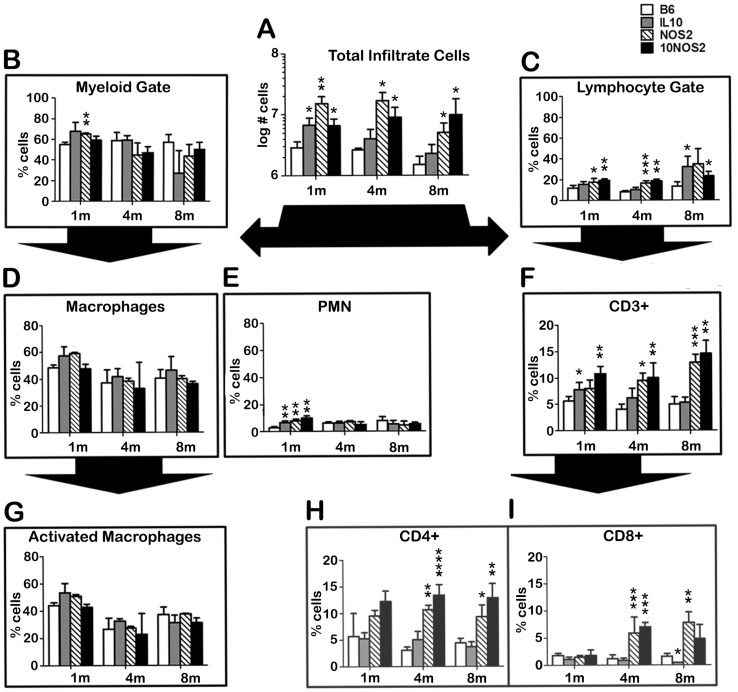
Immune cell populations in the *M. leprae*-infected FP. B6 (white bars), IL-10−/− (gray bars), NOS2−/− (striped bars), and 10NOS2−/− (black bars) FP were inoculated with 3×10^7^ *M. leprae*. At 1, 4 and 8 months post-infection, FP tissue was digested and the single cell suspensions were (A) counted, separated into (B) myeloid or (C) lymphoid gates based on side and forward scatter by flow cytometry, and categorized via staining with fluorescently labeled antibodies for (D) macrophages (CD11b+/Ly-6G−), (E) neutrophils (CD11b+/Ly-6G+), (G) activated macrophages (CD11b+/Ly-6G−/IAb+), (F) CD3+ T cells, (H) CD3+CD4+ T cells or (I) CD3+CD8+ T cells. A total of 15,000 cells per mouse were analyzed and results are expressed as percent of the indicated populations per foot pad. Experiment shown is representative of two independent experiments. *p≤0.05; **p≤0.01; ***p≤0.001; ****p≤0.0001.

Single cell suspensions were prepared from the FP tissues of each group during the three phases of infection. As shown in Fig. 5A, on the order of 10^6^ cells were recovered from B6 FP at each stage of infection. All knockout strains had significantly more cells infiltrating the FP at 1 month compared to B6 mice, and NOS2−/− and 10NOS2−/− maintained higher numbers of infiltrating cells into the peak and chronic stages. Cellular phenotypes at the site of infection determined by flow cytometry showed that ∼50–70% of the recruited cells were found in the myeloid gate (Fig. 5B) and ∼20–40% segregated to the lymphoid gate in all strains of mice (Fig. 5C).

Throughout the infection period, the majority of the myeloid cells in the FP from all strains was macrophages (Fig. 5D) and expressed IA^b^ (Fig. 5G). Neutrophils accounted for <10% of the FP cells (Fig. 5E). In the lymphoid gate, B cells and NK cells comprised <2% of the FP cells (data not shown). Major differences were seen, however, in the T cell populations (Fig. 5F) where NOS2−/− and 10NOS2−/− FP yielded a significantly increased percentage of CD3+ cells. In all strains, CD4+ cells (Fig. 5H) comprised the majority of the CD3+ cell population as compared to CD8+ cells (Fig. 5I). Both CD4+ and CD8+ cells, however, were augmented in the NOS2−/− and 10NOS2−/− FP, especially during the peak and chronic phases. As previously reported for B6 mice [14], the majority of the CD4+ and CD8+ cells infiltrating the FP in all strains expressed the activation phenotype of CD44+CD62L− (data not shown).

### IFN-γ generation by lymphocytes from the site of *M. leprae* infection

**Figure 6:**
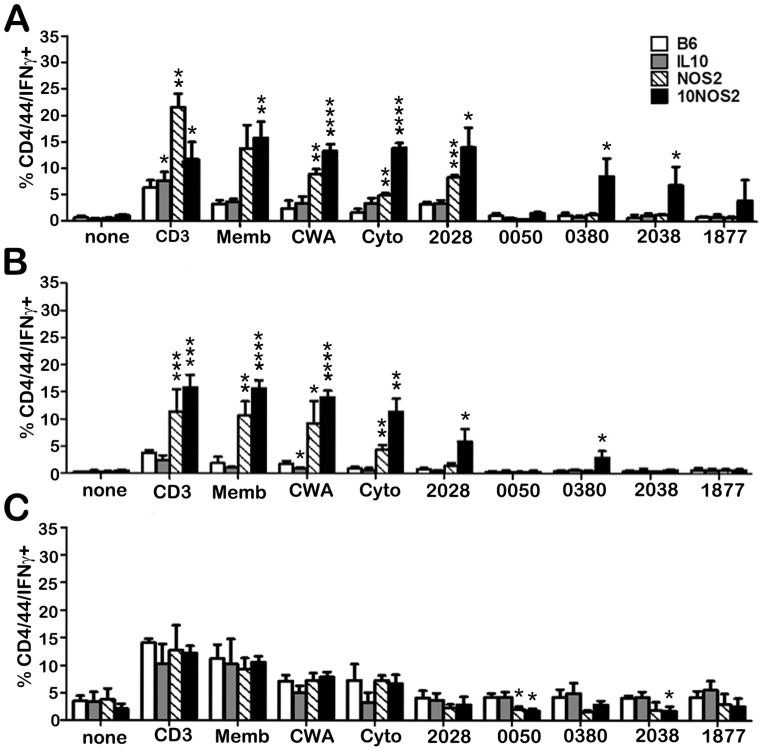
IFN-γ responses to *M. leprae* antigens by infiltrating CD4+CD44+ cells in the infected FP. B6 (white bars), IL-10−/− (gray bars), NOS2−/− (striped bars), and 10NOS2−/− (black bars) FP were inoculated with 3×10^7^ *M. leprae*. At (A) 1, (B) 4 and (C) 8 months post-infection, FP tissue was digested and the non-adherent cells were plated at 2×10^5^ cells/well and incubated with PBS, CD3 antibody, or *M. leprae* antigens (10 µg/ml) for 48 hrs. The cells were stained for CD4, CD44 and IFN-γ and analyzed by flow cytometry. A total of 10,000 cells per group were analyzed. Experiment shown is representative of two independent experiments. *p≤0.05; **p≤0.01; ***p≤0.001; ****p≤0.0001.

**Figure 7:**
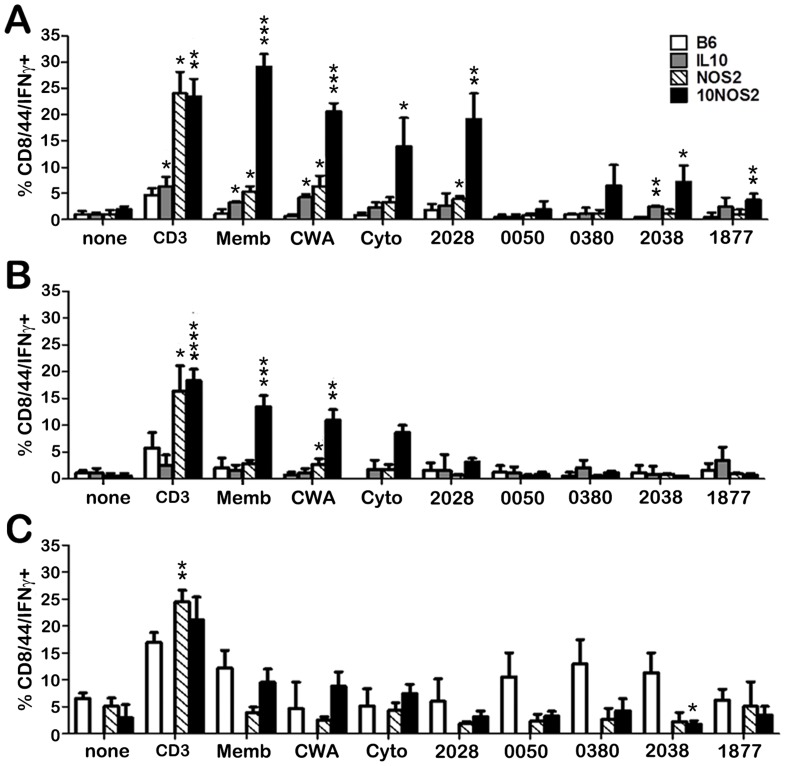
IFN-γ responses to *M. leprae* antigens by infiltrating CD8+CD44+ cells in the infected FP. B6 (white bars), IL-10−/− (gray bars), NOS2−/− (striped bars), and 10NOS2−/− (black bars) FP were inoculated with 3×10^7^ *M. leprae*. At (A) 1, (B) 4 and (C) 8 months post-infection, FP tissue was digested and the non-adherent cells were plated at 2×10^5^ cells/well and incubated with PBS, CD3 antibody, or *M. leprae* antigens (10 µg/ml) for 48 hrs. The cells were stained for CD8, CD44 and IFN-γ and analyzed by flow cytometry. A total of 10,000 cells per group were analyzed. Experiment shown is representative of two independent experiments. *p≤0.05; **p≤0.01; ***p≤0.001; ****p≤0.0001.

In order to assess the nature of the T cells recruited to the site of infection, FP granuloma lymphocyte populations from each strain were stimulated with various *M. leprae* crude and purified antigens in vitro and evaluated by flow cytometry for IFN-γ production by CD4+CD44+ (Fig. 6) and CD8+CD44+ (Fig. 7) cells. In general, all strains demonstrated an IFN-γ response by CD4+CD44+ (Fig. 6) and CD8+CD44+ (Fig. 7) cells to the crude *M. leprae* antigens (membrane, CWA, cytosol) and to ML2028 (Ag85B) at one or more points during long-term infection. In comparison to B6, NOS2−/− and 10NOS2−/− mice exhibited a significantly higher percentage of CD4+CD44+ and/or CD8+CD44+ cells that recognized these antigens. *M. leprae* membrane antigen evoked the strongest response of all the antigens screened. It was detected by 10NOS2−/− in 15.8±3.1% of CD4+CD44+ (*p* = 0.0041) at 1 month post infection, a response similar to that seen in NOS2−/− FP cells (Fig. 6A). The effect of both IL-10 and NOS2 deficiencies in recognizing the crude antigens was even more sharply demonstrated in CD8+CD44+ cells of 10NOS2−/− (Fig. 7A). *M. leprae* membrane antigen was recognized by 29.1±2.4% of CD8+CD44+ cells (*p* = 0.0002) which was a 5–6 fold increase over all other strains, including the NOS2−/− cells (*p*<0.0001).

Interestingly, the 10NOS2−/− FP T cells also exhibited an enhanced response to the purified antigens, ML0380, ML2038 and ML1877. ML2038 and ML1877 were recognized early by 10NOS2−/− CD4+CD44+ (Fig. 6A) and CD8+CD44+ (Fig. 7A) cells but not significantly at 4 months (Fig. 6B and 7B) or 8 months (Fig. 6C and 7C). ML0380 was recognized at 1 month (Fig. 6A) and at 4 months (Fig. 6B) by CD4+CD44+ cells. Control mice were not particularly responsive to ML0050, a finding previously reported in both *M. tuberculosis-* [43] and *M. leprae-* [5] infected B6 mouse models, and a lack of IL-10 and/or NOS2 did not enable responsiveness to this antigen.

## Discussion

Nerve damage in leprosy can be the consequence of immunological or inflammatory responses induced by the organism [11], [44], and the nerve injury seen at the tuberculoid end of the spectrum is thought to be due to destructive granulomatous inflammation. A variety of mechanisms have been proposed [45]: the *M. leprae*-infected Schwann cell can act as an antigen-presenting cell and signal destruction of the infected cells [46]; the inflammatory response can injure the nerve as an “innocent bystander”; a cell mediated immune response may become destructive when T cells necessary for the protective response induce tissue damage depending on the type and quantity of the local cytokine response, as during Type 1 reactions. We did not see infection of Schwann cells by *M. leprae* in the B6 or any of the knockout strains; hence, the first mechanism for nerve damage is unlikely in our model. While the second mechanism may play a role, the elevated IFN-γ response in the absence of IL-10 and appearance of T cells inside damaged nerves in the 10NOS2−/− FP would suggest the latter mechanism. Thus, a lack of IL-10 appears to result in a neuropathologic response in the inflammatory NOS2−/− model.

IL-10 up-regulation can suppress lymphocyte-driven immunity, and in leprosy IL-10 is detected in multibacillary lesions [47]–[50]. This *M. leprae*-induced IL-10 could contribute to the development of the T cell anergy seen at the Th2-dominant lepromatous end of the leprosy spectrum by steering the immune response toward a phagocytic rather than antimicrobial program [51]. In vitro, *M. leprae* infection stimulates monocytes to produce IL-10 [52]–[54], and IL-10 supplementation of infected macrophage cultures extends maintenance of *M. leprae* viability [55], [56]. In an experimental model, IL-10 expression is elevated in *M. leprae*-infected IFN-γ knockout mice which exhibit less resistance to infection compared to control mice [13].

Interestingly, IL-10 is also detected in paucibacillary leprosy lesions [48]. Its presence along with various Th1 cytokines has been suggested to be a mechanism for tempering immunopathology [57]. Iyer et al [58] proposed that the observation of both IL-10 and TNF in Type 1 and Type 2 reactions indicated the simultaneous suppression of proinflammatory pathways and activation of regulatory pathways to control tissue damage from excessive inflammation.

This study represents the first time IL-10 deficiency has been investigated 1) in vivo, 2) at the site of infection and 3) over acute and chronic infection in an experimental leprosy model. Similarities in *M. leprae* growth profiles across the strains suggest that an absence of IL-10, either alone or combined with a NOS2 deficiency, did not provide a more growth restrictive environment in the already highly resistant B6 strain. *M. leprae*-infected IL-10−/− mice exhibited slight exacerbations compared to B6 in histopathology, FP induration, and antigen responsiveness with no evidence of survival or resumed *M. leprae* growth over the course of infection. In contrast, IL-10−/− mice infected with BCG or *M. tuberculosis* exhibited high Th1 cytokine responses and enhanced inflammation with persistent granulomas containing reduced bacterial loads in short-term lung studies compared to wild type mice [59]–[62]. Nevertheless, a longer term study [63], demonstrated eventual morbidity due to *M. tuberculosis* regrowth.

In our studies with NOS2−/− mice ([17] and this paper), we found that *M. leprae* infection of the FP resulted in a large granuloma composed of numerous epithelioid cells and dense collections of lymphocytes. The granuloma infiltrated muscle bundles and partly destroyed them, with recruited cells exhibiting elevated levels of Th1 cytokines and chemokines, as well as IL-10; yet bacterial growth remained restricted as compared to B6. Similarly, previous studies using *M. avium* infection of NOS2 deficient mice have shown increased granuloma formation and cellularity and an up-regulation of Th1 cytokine, chemokine and IL-10 expression in the absence of bacterial replication [64], [65]. Thus, we questioned whether disruption of both IL-10 and NOS2 would exacerbate the NOS2 model further toward a more inflammatory or even “reactional” state. Infection of 10NOS2−/− FP with *M. leprae* resulted in a markedly enhanced accumulation of *M. leprae*-responsive CD4+ and CD8+ T cells in the granuloma and a CD4+ T cell infiltration into the local nerves, an outcome not seen in the single knockout mice nor previously reported in mouse models for leprosy.

It is noteworthy that peak inflammation developed within 3–4 months after *M. leprae* inoculation, a time when the bacteria have been killed. While ∼15% of reactions are identified at the time of leprosy diagnosis, reactions and neuropathy continue to occur during the first year of treatment (Scollard et al., submitted for publication) and even years after successful multidrug therapy (MDT) [66]–[68]. It has been hypothesized that antimicrobial therapy which kills the bacilli may cause release of increased or different antigens thereby heightening the risk for subsequent immunological complications in some patients. No *M. leprae*-specific antigens have yet been identified in relationship to this phenomenon.

Thus, the three induration phases revealed interesting contrasts among the mouse strains regarding which *M. leprae* antigens were recognized, which lymphocytes targeted them, and when they were targeted over the course of long term infection. In the 10NOS2−/− FP, there was a significantly stronger IFN-γ response to the crude antigens and ML2028 (Ag85B), as well as to ML0380 (GroES), ML2038 (bactoferritin), and ML1877 (EF-Tu) by both CD4+ and especially CD8+ T cells at the site of infection. Interestingly, the GroES, Ag85B, and EF-Tu antigens of *M. tuberculosis* have been reported to function as plasminogen receptors [69]. Activation of the mammalian plasminogen-plasmin system has been proposed to be a mechanism whereby pathogens can contribute to bacterial dissemination and tissue damage [70]. Whether these *M. leprae* homologues promote tissue damage via this pathway is an interesting prospect for investigation.

Despite 30 years of global MDT programs and redefined elimination targets, leprosy remains a persistently reported disease in a number of endemic countries. However, even if all current active cases of leprosy were detected and provided MDT today, a significant portion of those patients would still experience subsequent progressive disease complications for years to come due to immunological perturbations. Leprosy reactions are a major cause of permanent neuropathy and disability especially in patients with borderline disease. Our findings present the 10NOS2−/− strain as an interesting model for investigating lymphocyte recruitment into the granuloma and nerves throughout long term infection in the resistant forms of leprosy. Determining the antigens recognized by T cells at the site of *M. leprae* infection could significantly further understanding of immunopathogenesis. This in turn, could provide stepping stones for the development of *M. leprae*-antigen specific diagnostics to improve detection, monitoring, and treatment of leprosy patients.
